# Supplementary material for: Using the NIH Research, Condition and Disease Categorization Database for research advocacy: Schizophrenia research at NIMH as an example
Source: PLoS One. 2020 Nov 19;15(11):e0241062. doi: 10.1371/journal.pone.0241062 (PMC7676683; doi:10.1371/journal.pone.0241062)
Supplement: Attachment — Submitted filename: Response to Reviewers PLOS ONE.docx [file pone.0241062.s003.docx]

Dear Dr. Naudet, Dr. Minogue and Reviewer #2,

Thank you for your useful comments and critiques. We have done the following:

- We have stated explicitly that there was not a protocol.
- The master file, including all 428 research projects, by title and number along with the grades of the three reviewers are being submitted as supplementary material. To make the order of reviewers consistent between supplementary material and the manuscript, we reversed the numbers between reviewers 2 and 3 in Table 1.
- We have included a reporting checklist, along with mention in the methods section, a reference, and a specific grid.
- We have re-written the methods section to make explicit the exploratory nature of the study.
- We have also included this in the sections on limitations at the end of the paper.
- We have included three Venn diagrams to show the agreement among the three reviewers for the three questions.
- We have added Table 2 to include the full list of the 30 proposals selected as “likely”.
- The supplementary material now includes all studies in the same order as they are listed in the RCDC database. Thus individuals can ascertain the rating for all studies listed as “likely”, “possible”, or “very unlikely”.
- The Venn Diagrams are being submitted as separate figure files.
- The Title and Numbers of the two patents that were included pertain to depression and thus are not relevant to our study on schizophrenia. We have added the suggested statement.

Dr. Minogue

- In re-writing the methods section, we clarified the intent and development of the study, including the use of the advocacy groups in its development.
- The intended purpose of the RCDC database was to make the information available to the public and to shift the lobbying for funding from members of Congress to the NIH institutes. The directors of the advocacy groups were included as authors.
- We have added a section in the discussion regarding how the study will be used by the advocacy groups. Unfortunately we do not have an organization similar to the James Lind Alliance as far as I know. The main relationship between the NIH institutes and most disease advocacy groups is the former asking the latter to ask Congress to give them more money; thus what we are doing is very different.
- The methods section has been enlarged to provide more details on the development of the study. We have added “new and ongoing” to indicate that the projects from the past have been included.
- We have clarified that the ratings of “likely”, “possible”, and “very unlikely” were subjective based on individual experience and expertise. We did not attempt to standardize the definitions.
- We decided to use three assessors as a guess, based on the fact that we had 428 research projects. We had no idea when we started how much agreement we would have and were pleasantly surprised to find that we had good agreement.
- We added a sentence to clarify that our shared bias was that NIMH should be doing both basic and clinical research.
- Most of the project abstracts were sufficiently detailed to make a reasonable assessment of what they are likely to produce over the next twenty years.
- We have added an explanation for why twenty years was selected as the optimum period.
- In the abstract “criticisms” has been substituted for “allegations”.
- The final sentence in the abstract has been modified.
- Based on my discussion with the NIH official who was in charge of the RCDC until he recently retired, I think it is very unlikely that the database was used by any disease advocacy groups.
- The prevalence of schizophrenia in the US is probably a little above average by world standards. In most studies, it has been said to be one of the costliest diseases.
- We have spelled out NAMI and PET.
- We have significantly enlarged the description for the review process.
- We have enlarged the discussion of research projects that would be clinically useful that NIMH should be doing.

Reviewer #2

- We have clarified why we are using the 1983 prevalence data.
- Although the descriptions of some of the intramural research projects was sparse, the reviewers had independent information on many of them. For example, two of us had worked with researchers doing the imaging studies many years ago. Thus we felt comfortable in our ratings. The percentage of NIMH funds going to intramural projects is stated at the beginning of the results section.
- We added additional examples of the kind of schizophrenia research that NIMH should be funding.
