# Supplementary material for: Using the NIH Research, Condition and Disease Categorization Database for research advocacy: Schizophrenia research at NIMH as an example
Source: PLoS One. 2020 Nov 19;15(11):e0241062. doi: 10.1371/journal.pone.0241062 (PMC7676683; doi:10.1371/journal.pone.0241062)
Supplement: Attachment — Submitted filename: Response to reviewers.docx [file pone.0241062.s004.docx]

Florian Naudet, M.D., M.P.H., Ph.D.
Academic Editor
PLOS ONE

September 10, 2020

Dear Dr. Naudet,

Thank you for the further review of our manuscript. We have done the following.

- Clarified in the abstract that the conclusions are only exploratory;

- Clarified in the abstract the limitations of the research, including the limitations of the agreement among the raters;

- Stated explicitly in the text that the interrater agreement was good for the “very unlikely” research projects, but only fair for the “possible” projects and poor for the “likely” projects. This should also be clear to readers from looking at the Venn diagrams.

Regarding the proper statistical test for assessing the interrater reliability, my colleagues (who understand this issue much better than I do) believe that the Cronbach's alpha is the more appropriate test because it is a measure of internal consistency and scale reliability. What we are testing is scale reliability, not a diagnostic test or x-ray, etc. Cronbach’s is most commonly used for scale reliability and that seems consistent with what we are doing. Cohen`s kappa, by contrast, is usually used when you have just two raters. However if you prefer that we use a kappa statistic we can look for an alternative to the Cohen’s kappa.

I am also sad to report that one of our authors, DJ Jaffe, died unexpectedly on August 23. I have so indicated on the title page.

E. Fuller Torrey, M.D

Associate Director for Research

Stanley Medical Research Institute

Kensington, MD
