# Supplementary material for: Using the NIH Research, Condition and Disease Categorization Database for research advocacy: Schizophrenia research at NIMH as an example
Source: PLoS One. 2020 Nov 19;15(11):e0241062. doi: 10.1371/journal.pone.0241062 (PMC7676683; doi:10.1371/journal.pone.0241062)
Supplement: Attachment — Submitted filename: Response to Reviewers.docx [file pone.0241062.s005.docx]

RE: Manuscript **PONE-D-20-13102R2**
Using the NIH Research, Condition and Disease Categorization Database for research advocacy: Schizophrenia research at NIMH as an example

Florian Naudet, M.D., M.P.H., Ph.D.
Academic Editor. DATE: October 5, 2020
PLOS ONE

Dear Dr. Naudet,

Following up your letter of September 11, we have consulted with Thomas Carmody PhD, a Professor in the Department of Population and Data Sciences at the University of Texas Southwestern. After reviewing our manuscript and some of the literature on interrater reliability, he concluded that both Fliess kappa and Cronbach alpha have been used in situations analogous to ours. He sent a couple of references justifying the use of the latter. However, he also said that the kappa “is more widely used for interrater agreement”, whereas alpha “is most often used to measure internal consistency of scales”. We, therefore, decided to use the Fliess kappa which he calculated for us:

**Fleiss kappa for each level of rating and overall**

| Rating | Kappa | Error | z | Prob > z |
| --- | --- | --- | --- | --- |
| 1 | 0.16513 | 0.027907 | 5.9171 | <.0001 |
| 2 | 0.42658 | 0.027907 | 15.2858 | <.0001 |
| 3 | 0.56954 | 0.027907 | 20.4083 | <.0001 |
| overall | 0.47261 | 0.024540 | 19.2588 | <.0001 |

We have made the appropriate changes to the text.

Thank you for your assistance.

Please let us know if you need additional information.  

E. Fuller Torrey MD
